# Electroconvulsive therapy reduces suicidality and all-cause mortality in refractory depression: A systematic review and meta-analysis of neurostimulation studies

**DOI:** 10.1016/j.nsa.2025.105520

**Published:** 2025-06-02

**Authors:** Jolein Odermatt, Jan Sarlon, Neysan Schaefer, Sarah Ulrich, Magdalena Ridder, Else Schneider, Undine E. Lang, Timur Liwinski, Annette B. Brühl

**Affiliations:** University Psychiatric Clinics Basel, Clinic for Adults, University of Basel, Basel, Switzerland

**Keywords:** Depression, Major depressive disorder, Bipolar disorder, Electroconvulsive therapy, Repetitive transcranial magnetic stimulation, Suicidality, Suicide, Mortality

## Abstract

Depressive disorders are among the most common psychiatric disorders worldwide and associated with half of all suicides. There is robust evidence indicating that both electroconvulsive therapy (ECT) and repetitive transcranial magnetic stimulation (rTMS) effectively alleviate depressive symptoms in difficult-to-treat depression and enhance patient outcomes. However, there remains ongoing debate regarding their potential roles in preventing suicide and reducing all-cause mortality. Our study aims to investigate the impact of various neurostimulation techniques, including ECT, rTMS, and vagus nerve stimulation (VNS), on reducing suicidality, including suicidal ideation and completed suicides, as well as on overall mortality among individuals diagnosed with depression. In this systematic review and meta-analysis, we searched on MEDLINE via PubMed until January 9, 2024 for randomised controlled trials and controlled observational studies that investigated suicide and all-cause mortality outcomes after neurostimulation treatment for depression.

Of the 1351 screened records we identified 26 studies eligible for inclusion in our systematic review. We included 11 studies on ECT (involving 17′890 subjects treated with ECT and 25′367 controls receiving treatment as usual), 5 studies on rTMS and 3 studies on VNS in our meta-analysis. In the cumulative cohort, 208 suicide deaths (1.70 %) were observed in the ECT group and 988 suicide deaths (5.02 %) were registered in the control group. Moreover, there were 511 deaths from all causes (3.13 %) in the ECT group, compared to 1325 deaths (6.64 %) in the control group. Thus, treatment with ECT demonstrated a significant 34 % decrease in the odds of suicide (OR 0.66, 95 % CI 0.50–0.88, p = 0.0047) and a 30 % reduction of death from all causes (OR 0.70, 95 % CI 0.62–0.79, p < 0.0001). The standardized mean difference (SMD) for suicidal ideation before and after ECT was −0.58 (95 % CI –0.10 to −1.07, p = 0.0177), suggesting a moderate effect size. We found no significant effect of rTMS on suicidal ideation with an SMD of −0.41 (95 % CI –1.01 – 0.19, p = 0.1795). In patients treated with VNS a 60 % reduction in the odds of death from all causes was observed (OR 0.40, 95 % CI 0.18–0.92, p = 0.0306).

To conclude, there is consistent observational data supporting the protective effects of ECT against suicide and overall mortality.

The systematic review protocol is registered online on PROSPERO, CRD42023412887.

## 1 Introduction

Major depressive disorder (MDD) is among the most common psychiatric disorder with an estimated number of 300 million people affected worldwide. This corresponds to 4.3 % of the global population. Moreover, the number of individuals with depression has increased by almost 20 % from 2005 to 2015 (WHO, 2017). Depression is defined by periods of depressed mood or reduced pleasure or interest lasting for at least two weeks (WHO, 2023). Depression is closely associated with suicide, representing the most prevalent psychiatric disorder among individuals who die by suicide (Hawton et al., 2013). With over 700,000 suicide fatalities annually, suicide stands as the fourth leading cause of death among young adults aged 15–29 years (WHO, 2023). In the year 2020, the European Union documented 47,252 fatalities attributed to deliberate self-inflicted harm, accounting for nearly 1 % of all reported deaths during that period (Eurostat, 2024). While suicide itself is not classified as a disease, suicidal behaviour encompassing both completed suicides and suicide attempts represents a significant public health concern. Consequently, it has garnered escalating research interest and has been the focal point of public awareness initiatives over the last decade. Moreover, it stands as a prominent area of focus within the global public health community (World Health Organization, 2014). Every suicide fatality is a unique tragedy and is projected to have significant repercussions on numerous individuals, encompassing family members, friends, and communities (Cerel et al., 2019). Half of all suicides are associated with depressive and other mood disorders classified under ICD-10 F3. Compared to individuals without such conditions, there is a reported twentyfold elevation in the risk of suicide (Osby et al., 2001; Lépine and Briley, 2011). In collaboration with the National Action Alliance for Suicide Prevention (NAASP), the National Institute of Mental Health (NIMH) is dedicated to achieving a 20 % reduction in the suicide rate by the year 2025 (Gordon et al., 2020). However, despite these efforts, suicide rates in the United States surged by 37 % between 2000 and 2018, and exhibited a subsequent decline of 5 % between 2018 and 2020. Nevertheless, rates almost reverted to their peak in the year 2021 (Centers for Disease Control and Prevention (CDC), 2023). Recently published data from the US Centers for Disease Control and Prevention shows that the current suicide rate in the US has reached its highest recorded value in >70 years, as the last time higher numbers were observed was in 1941 (National Center for Health Statistics, 2019).

Treatment-resistant depression (TRD) is conventionally defined as the absence of remission following the appropriate administration of two or more antidepressants. Expanding on the TRD concept, difficult-to-treat depression (DTD) delineates a clinical scenario where individuals fail to achieve full alleviation of symptoms despite undergoing various therapeutic interventions (Paganin, 2023). Previous estimations indicate that roughly one-third of individuals diagnosed with depression demonstrate resistance to treatment (Zhdanava et al., 2021; McIntyre et al., 2014). However, the efficacy of antidepressants may be lower than previously assumed, as evidenced by a cumulative remission rate of 35.0 % following up to four antidepressant treatment trials (Pigott et al., 2023). Importantly, there remains a lack of evidence from randomized controlled trials (RCTs) supporting the efficacy of antidepressants in preventing suicides and suicide attempts (Braun et al., 2016). In contrast to depressed individuals lacking treatment resistance, patients with treatment-resistant depression encounter not only a heightened risk of suicide but also an increased risk of all-cause mortality (Brenner et al., 2021; Li et al., 2019). In light of the public health crisis surrounding depression and suicide, there exists a critical necessity for evidence supporting the efficacy of various treatment modalities (Mann et al., 2021; Griffiths et al., 2014). Such approaches should demonstrate clear effectiveness in addressing both death by suicide and overall mortality endpoints (Ahrens, 1994). Neurostimulation procedures are promising therapeutic options based on electrical or magnetic stimulation of the brain, e.g. electroconvulsive therapy (ECT), repetitive transcranial magnetic (rTMS) and vagus nerve stimulation (VNS) (Carpenter, 2006; Kennedy et al., 2009). These techniques are typically employed in clinical settings following the establishment of resistance to pharmaceutical antidepressant treatment (Park et al., 2015).

ECT was first used in 1938 (Kennedy et al., 2009). During ECT, small electrical current pulses between 0.5 and 0.9 A are applied to trigger seizures, thereby altering the concentration of neurotransmitters like GABA and monoamines such as norepinephrine, serotonin, and dopamine. Furthermore, it is suggested that ECT may influence the neuroplasticity of the cortical system (Kennedy et al., 2009; Merkl et al., 2009; Espinoza Randall and Kellner Charles, 2022). Comparative investigations have unequivocally demonstrated that ECT exhibits a superior antidepressant efficacy in comparison to pharmacotherapeutic agents (Heijnen et al., 2010; Dierckx et al., 2012). ECT is usually performed in two or three sessions per week under general anaesthesia with muscle relaxation (Kennedy et al., 2009). Several meta-analyses show a greater reduction of depressive symptoms for ECT compared to sham ECT or pharmacotherapy (UK ECT Review Group, 2003; Pagnin et al., 2004; Kho et al., 2003). The observed low incidence rate of 0.097 % for serious potentially life-threatening adverse events (pLTAEs) necessitating medical intervention suggests the potential for considering ECT as a relatively safe treatment option when conducted within a controlled environment (Hajak et al., 2021; Tørring et al., 2017). In specific circumstances, ECT might present a safer option compared to alternative pharmacological treatments, particularly for elderly individuals, pregnant women, and those who are physically debilitated (American Psychiatric Association, 2008).

rTMS shows favourable results with a lower incidence of reversible cognitive impairment than ECT (McIntyre et al., 2014). A recent meta-analysis from 2023 reveals rTMS on the left dorsolateral prefrontal cortex to be significantly more effective at reducing depressive symptoms compared to sham rTMS (Kan et al., 2023). rTMS is a non-invasive procedure where an electrical current in the brain is induced by magnetic pulses which leads to a stimulation of neurons and neuronal networks (Hallett, 2000). Vagus nerve stimulation (VNS) is performed by a pulse generator implanted into the patient's chest wall and electrodes wrapped around the left cervical vagal nerve in the neck area (George et al., 2000). Other types of neurostimulation include a variant of ECT called Magnetic Seizure therapy (MST), Transcranial Direct Current Stimulation (tDCS) or Deep Brain Stimulation (DBS) (De Raedt et al., 2015).

Although RCTs have demonstrated the antidepressant efficacy of ECT and rTMS, there remains an ongoing debate regarding their potential roles in preventing suicide and reducing all-cause mortality (UK ECT Review Group, 2003; Kan et al., 2023). In response to concerted endeavours aimed at mitigating the escalating incidence of suicide, we undertook a systematic review and meta-analysis to assess the efficacy of established interventions proven to diminish suicide risk. Our study aims to investigate the impact of neurostimulation techniques on mitigating suicidality, encompassing suicidal ideation, suicide attempts, and completed suicides, as well as on overall mortality rates among individuals diagnosed with depression.

## 2 Methods

### 2.1 Systematic review and selection criteria

We carried out a systematic review and meta-analysis to assess the evidence from human randomised controlled trials and observational studies (including cohort and case-control studies) for associations between neurostimulation and suicidality and mortality in depression. Uncontrolled studies, reviews and case reports were excluded. We registered the protocol with PROSPERO (registration identification CRD42023412887). This systematic review and meta-analysis were conducted in adherence to the Preferred Reporting items for Systematic Review and Meta-analysis (PRISMA) guidelines (Page et al., 2021).

We defined inclusion and exclusion criteria based on the Population, Intervention, Comparison, and Outcome (PICO) framework (Huang et al., 2006). Studies must satisfy all of the following inclusion criteria in order to be included.

• Population: Studies including human individuals diagnosed with unipolar or bipolar depression.
• Intervention: Neurostimulation procedures including electroconvulsive therapy (ECT), Transcranial magnetic stimulation (TMS), including its various modalities—most notably repetitive transcranial magnetic stimulation (rTMS) and (intermittent) theta burst stimulation (iTBS), which is generally considered a variant of rTMS (Huang et al., 2005), vagus nerve stimulation (VNS), transcranial direct current stimulation (tDCS), deep brain stimulation (DBS), magnetic seizure therapy (MST), epidural stimulation, trigeminal nerve stimulation (TNS), low field magnetic stimulation (LFMS), transcranial static magnetic field stimulation (tSMS), transcranial focused ultrasound (tFUS), and neurofeedback.
• Comparison: Studies with a comparison of neurostimulation versus standard of care therapy.
• Outcome: Studies reporting data on suicidality including suicidal ideation, suicide attempts and completed suicides, or all-cause mortality.

Studies meeting one of the following criteria were excluded.

• Population: Animal studies and studies restricted to individuals with other diagnoses.
• Intervention: Studies without a neurostimulation intervention.
• Comparison: Studies without comparison of neurostimulation and standard of care therapy.
• Outcome: Studies without a reported outcome mentioned above.

### 2.2 Search strategy

We performed a systematic search on MEDLINE via PubMed to January 9, 2024, with no language restriction. We used the following search terms:

(depression OR depressed OR major depression OR major depressive disorder OR MDD OR dysthymia OR treatment-resistant depression OR TRD OR difficult-to-treat depression OR DTD)

AND.

((electroconvulsive therapy OR ECT) OR (transcranial magnetic stimulation OR TMS OR repetitive transcranial magnetic stimulation rTMS OR theta-burst stimulation OR intermittent theta-burst stimulation OR TBS OR iTBS) OR (vagus nerve stimulation OR VNS) OR (transcranial direct current stimulation OR tDCS) OR (deep brain stimulation OR DBS) OR (magnetic seizure therapy OR MST) OR (epidural stimulation) OR (trigeminal nerve stimulation OR TNS) OR (low field magnetic stimulation OR LFMS) OR (transcranial static magnetic field stimulation OR tSMS) OR (transcranial focused ultrasound OR tFUS) OR (neurofeedback))

AND.

((suicide OR suicidality OR suicidal ideation OR suicide attempt OR self-murder OR self-slaughter OR self-harm OR self-injury) OR (mortality OR death OR all-cause mortality))

If there were any previous relevant citations in articles not detected in the initial search, they were screened for eligibility as part of forward citation searching.

### 2.3 Study selection and data extraction

Titles and abstracts were screened, and potentially relevant articles underwent full-text eligibility assessment by two independent authors. The outcomes of interest were extracted from each study and manually transferred into the results table. If studies had not reported all necessary data, unpublished results were requested by email from the corresponding authors. We also performed a manual search of the reference lists in the included articles. Some of the included articles were based on the same original study or cohort. When outcomes of interest were reported in multiple publications using the same study population, we included data from the publication with the largest sample size to avoid double counting (Witt et al., 2013). All analyses in the current work were thus conducted at the level of independent samples, rather than at the level of individual publications.

### 2.4 Statistical analysis

The analysis was carried out using R (version 4.3.2) (R Core Team, 2020) primarily leveraging the metafor package (version 4.4.0) (Viechtbauer, 2010), unless specified otherwise. Random effects models were utilized to estimate aggregated effect sizes. Binary outcome analyses pertaining to suicide and all-cause mortality events were conducted utilizing the log odds ratio (OR) as the primary outcome measure, with ORs reported alongside their corresponding confidence intervals (CIs) to facilitate interpretation. For suicidal ideation measured as a continuous variable using standardized instruments, we calculated Cohen's d, representing the standardized mean difference (SMD). This was carried out using the formula *d = (M*_*Intervention*_ - *M*_*Control*_*)/SD*_*pooled*_, where *M* represents the respective means of measurements for the intervention and active control groups, and *SD*_*pooled*_ the pooled standard deviation. By default, the metafor package adjusts Cohen's d for potential positive bias in small samples.

We employed the ‘find.outliers’ function from the dmetar package to detect outliers (Harrer et al., 2019). This function identifies studies as outliers if their 95 % confidence interval falls outside the 95 % confidence interval of the pooled effect. Additionally, it recalculates the meta-analysis results after excluding outliers.

The *I*^*2*^ statistic was employed to quantify heterogeneity, with 25 % indicating low, 50 % moderate, and 75 % high heterogeneity (Harrer et al., 2021). Additionally, the Q test was conducted to assess statistical significance. Studentized residuals and Cook's distances were utilized to assess the influence of individual studies within the model. Studies with Cook's distances surpassing the median plus six times the interquartile range of the Cook's distances were deemed to be disproportionally influential. The Excess Significance Test was employed to determine whether the number of studies reporting nominally significant results (p < 0.05) exceeded what would be expected based on statistical power. Additionally, Egger's regression asymmetry test was utilized to identify potential publication bias and small study effects, as smaller studies may frequently produce different, and often larger, effect estimates compared to their larger counterparts.

### 2.5 Assessment of study quality and risk of bias

A quality assessment of the included non-randomised studies was performed using the Newcastle-Ottawa Scale (NOS) (Wells et al.,). Additionally, risk of bias was assessed with the Risk of Bias In Non-randomized Studies (ROBINS-I) tool (Sterne et al., 2016) respectively the revised Cochrane risk-of-bias tool for randomized trials (RoB2) (Sterne et al., 2019). The Grading of Recommendations Assessment, Development and Evaluation (GRADE) methodology was utilized for rating the certainty of evidence (Guyatt et al., 2008). The quality of evidence for each pooled analysis was classified as "high," "moderate," "low," or "very low" (Supplementary Table 1). The GRADE approach initially classifies all observational studies as low-quality evidence. Of the eight criteria in the GRADE method, five can reduce confidence in effect estimates—risk of bias, inconsistency, indirectness, imprecision, and publication bias. Three criteria, however, can increase confidence: a substantial effect size without plausible confounders, a dose-response relationship, and residual confounding that would support the effect of exposure (Kirmayr et al., 2021). When applying GRADE to dichotomous outcomes such as mortality, no predefined thresholds for minimally important differences are established. However, an odds ratio reduction of ≤0.75, with both ends of the 95 % confidence interval below this threshold, is recommended (Kirmayr et al., 2021). We adopted this criterion in our assessment. For continuous outcomes, the clinical minimally important difference (MID) is a key determinant. Due to the heterogeneity of suicide rating scales and the lack of a well-defined MID for these measures, our criterion was that the upper limit of the 95 % confidence interval should be SMD≤ −0.2, corresponding to a reduction in suicidal ideation with at least a small effect size. We conducted a credibility assessment of the evidence using data from our analyses, including P values, I^2^ statistics, 95 % prediction intervals, small study effects, excess significance bias, and the significance of the largest study. Each pooled analysis was categorized as convincing (“Class I″), highly suggestive (“Class II”), suggestive (“Class III”), weak (“Class IV”), or lacking evidence (“Class V″) according to established classification criteria and prior systematic reviews and meta-analyses (Veronese et al., 2020; Huang et al., 2023; Belbasis et al., 2015; Fusar-Poli and Radua, 2018; Lane et al., 2024) (Supplementary Table 2).

## 3 Results

**Fig. 1:**
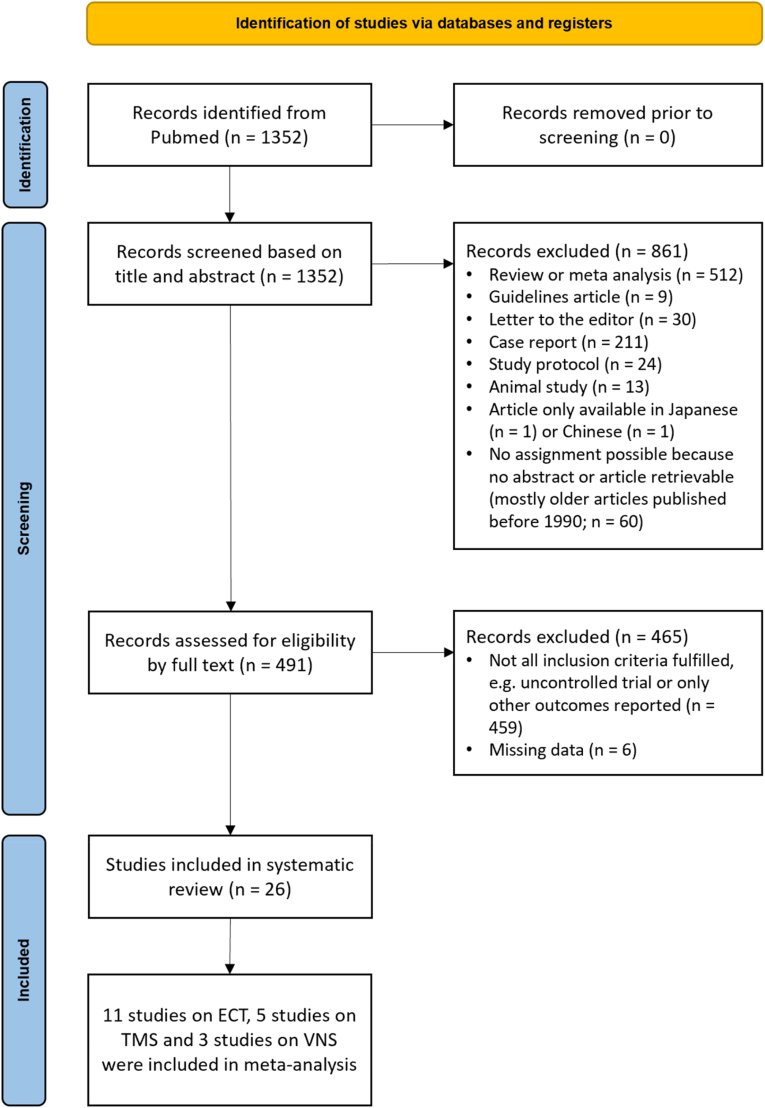
Study selection diagram (studies published between July 1, 1963 and January 3, 2024).

Of the 1352 screened records we identified 26 studies eligible for inclusion in this review. We identified 15 eligible studies on ECT, seven on rTMS, three on VNS and one on tDCS. We included 19 studies in the meta-analysis. A PRISMA flow diagram of the detailed study selection is shown in Fig. 1. Detailed information on all included studies is provided in Table 1 (numerical summary) and Supplementary Table 3 (descriptive summary).

**Table 1:** Summary of all studies included in systematic review.

	Total number of participants		Suicide		Deaths by all cause		Suicide thoughts baseline		Suicide thoughts after intervention		Suicide thoughts reduction	
Study	EG	CG	EG	CG	EG	CG	EG	CG	EG	CG	EG	CG
**ECT**												
Avery et Winokur (1976)	257	262	4	4	11	21	–	–	–	–	–	–
*Babigian et Guttmacher (1984)*	818.3	3109.6	18	58	243	470	–	–	–	–	–	–
*Brådvik et Berglund (2000)*	49	129	23	66	–	–	–	–	–	–	–	–
Nordenskjöld et al. (2013)	28	28	0	1	–	–	–	–	–	–	–	–
*Ahmadi*et al. *(2016)*	92	3393	2	201	9	612	–	–	–	–	–	–
*Liang*et al. *(2018)*	487	1948	72	515	–	–	–	–	–	–	–	–
Jørgensen et al. (2020)	5004	87891	103	495	809	11409	–	–	–	–	–	–
*Kheirabadi*et al. *(2020)*^a^*→ ECT vs. Ketamine p.o.*	12	12	–	–	–	–	10.75 ± 5.61	8 ± 4.19	4.25 ± 4.45	3.75 ± 2.9	–	–
*Kheirabadi*et al. *(2020)*^a^*→ ECT vs. Ketamine i.m.*	12	15	–	–	–	–	10.75 ± 5.61	8.1 ± 5.61	4.25 ± 4.45	3.13 ± 2.55	–	–
*Lin*et al. *(2020)*^b^	111	114	–	–	–	–	2.8 ± 0.8	2.5 ± 6.8	0.3 ± 0.8	1.1 ± 1.1	–	–
Rönnqvist et al. (2021)	5525	5525	62	90	161	237	–	–	–	–	–	–
Kaster et al. (2021)	5008	5008	5	11	16	23	–	–	–	–	–	–
*Nordenskjöld*et al. *(2022)*	5476	5476	–	–	192	258	–	–	–	–	–	–
Salagre et al. (2022)	11556	55269	144	97	–	–	–	–	–	–	–	–
Kaster et al. (2022)	4982	5304	27	54	138	197	–	–	–	–	–	–
*Cai*et al. *(2023)*^c^	81	79	–	–	–	–	18.57 ± 2.85	18.97 ± 3.03	3.70 ± 1.96	6.44 ± 1.78	–	–
**rTMS**												
George et al. (2014)	20	21	0	0	–	–	21.7 ± 5.7	20.8 ± 5.3	–	–	−15.6	−15.3
Desmyter et al. (2016)	14	18	–	–	–	–	13.43 ± 7.30	13.22 ± 7.47	9.36 ± 8.49	7.33 ± 8.68	–	–
*Yesavage*et al. *(2018)*^a^	81	83	0	0	0	0	3.7 ± 6.0	5.6 ± 6.7	1.5 ± 4.2	2.5 ± 4.9	–	–
*Dai*et al. *(2020)*^c^	48	55	–	–	–	–	17.60 ± 3.68	16.75 ± 3.36	9.85 ± 2.08	10.60 ± 2.68	–	–
Pan et al. (2020)	21	21	–	–	–	–	19.00 ± 5.94	21.48 ± 5.91	–	–	−14.76 ± 7.22	−4.71 ± 5.30
Pan et al. (2023)	31	28	–	–	–	–	17.50 ± 2.67	17.25 ± 2.22	–	–	−12.94 ± 8.46	−4.29 ± 4.89
Zhao et al. (2023)^a^	23	22	–	–	–	–	44.52 ± 5.08	44.86 ± 5.15	40.43 ± 5.86	44.27 ± 5.26	−4.08 ± 7.19	−0.59 ± 3.59
**VNS**												
*Olin*et al. *(2012)*	335	301	1	1	5	5	–	–	–	–	–	–
Feldman et al. (2013)	690	12163	–	–	37	2396	–	–	–	–	–	–
*Aaronson*et al. *(2017)*	494	301	2	2	7	8	–	–	–	–	–	–
**tDCS**												
Chen et al. (2023) → 60-min tDCS sessions vs. sham treatment	22	16	–	–	–	–	24.66 ± 9.93	28.75 ± 10.52	21.31 ± 6.15	18.38 ± 9.40	–	–
Chen et al. (2023)*→ 30-min tDCS sessions vs. sham treatment*	25	16	–	–	–	–	28.12 ± 7.86	28.75 ± 10.52	19.62 ± 8.51	18.38 ± 9.40	–	–

EG: Experimental group, CG: Control group.

ECT: Electroconvulsive therapy, rTMS: Repetitive transcranial magnetic stimulation, VNS: Vagus nerve stimulation.

a Suicidal ideation was measured using the Beck Scale for Suicidal Ideation (BSSI).

b Suicidal ideation was measured using the HAMD-17 suicide item (item 3).

c Suicidal thoughts were measured using the Self-rating Idea of Suicide Scale (SIOSS).

### 3.1 Study quality and risk of bias

**Fig. 2:**
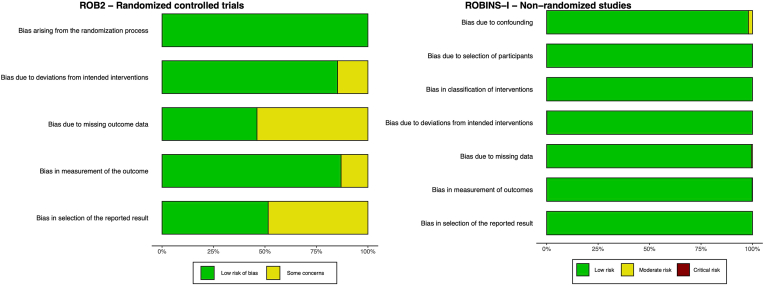
Summary of risk of bias assessment using the ROB2- and ROBINS-I-tool.

The detailed study quality assessment is provided in Supplementary Table 1. We considered all included non-randomized studies to be of good quality and most included studies had an overall low risk of bias (Fig. 2). A few randomized controlled trials showed some concerns mostly because of bias due to missing outcome data or bias in selection of the reported results because there was no pre-specified analysis plan available. One study (Brådvik and Berglund, 2000) was rated ‘critical’ due to selection bias since participants who died by suicide were matched with patients without a suicidal event.

### 3.2 Electroconvulsive therapy

The meta-analysis included 11 studies involving 17′890 subjects treated with ECT and 25′367 controls receiving treatment as usual (TAU). Follow-up time varied from a minimum of 30 days up to 9 years. Outcomes included suicide events, suicidal thoughts measured with standardised instruments, and all-cause mortality. Suicidal thoughts were measured by the Beck Scale for Suicidal Ideation (BSSI), the HAMD-17 suicide item (item 3) or the Self-rating Idea of Suicide Scale (SIOSS).

We excluded certain studies from the meta-analysis either due to scarcity of events (zero versus one suicide events) (Nordenskjöld et al., 2013) or because of considerable overlap of identical patient data between publications (Kaster et al., 2021, 2022). In this case, the most recent and more comprehensive publication was included (Kaster et al., 2022). We excluded Jørgensen et al. (2020) (Jørgensen et al., 2020) because the indication for ECT was not clear, resulting in patients with mild depression reportedly also receiving ECT. This discrepancy does not align with clinical practice or guideline recommendations, which typically reserve ECT for patients with severe as well as chronic or difficult-to-treat depression (Thirthalli et al., 2023; National Institute for Health and Care Excellence NICE, 2022; Bundesärztekammer (BÄK) et al., 2022). As the authors themselves note, ECT is typically not offered to patients with mild depression. They attribute the inclusion of such patients not to maintenance treatment but to "diagnostic uncertainty" and acknowledge "residual confounding and lack of precision" (Jørgensen et al., 2020). These factors further reinforced our decision to exclude this study from the meta-analysis. Nevertheless, in this study, ECT treatment was correlated with reduced all-cause mortality, and patients with the most severe depression exhibited the lowest relative risk for re-hospitalization, attempted suicide, and completed suicide compared to non-ECT controls (Jørgensen et al., 2020). Salagre et al. (2022) (Salagre et al., 2022) used a mirror-image-analysis instead of a direct comparison. However, in this study, significant and notable reductions were observed in the incidence of self-harm/suicide attempts when comparing the month preceding ECT initiation to the month following it. Additionally, those two studies were also identified formal outliers. We excluded Babigian et Guttmacher (1984) in the analysis of all-cause mortality because it was uncovered as a formal outlier (Babigian and Guttmacher, 1984). Another issue arose as this study did not compare matched cohorts. An examination of the diagnostic and demographic characteristics between the ECT and non-ECT first-hospitalization groups revealed significant differences across all relevant variables, including disease severity, the ECT group being more severely affected. Suicide rates and all-cause mortality were comparable between the two groups.

#### 3.2.1 ECT and suicide

**Fig. 3:**
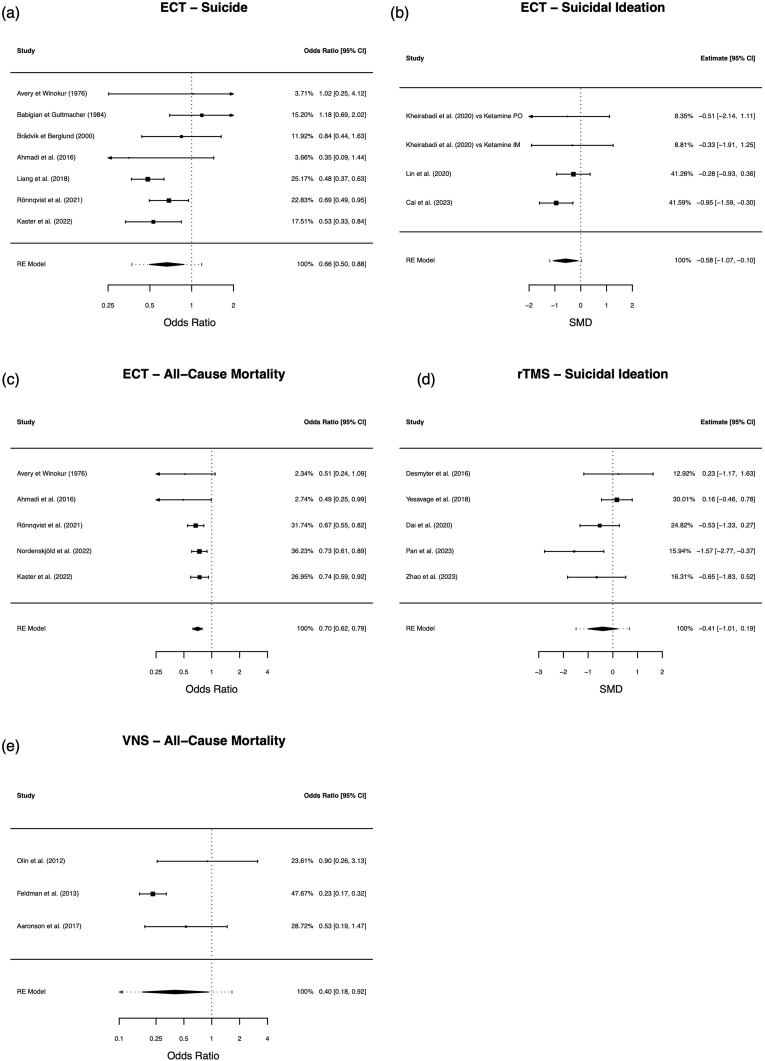
Forest pot summarizing the study's meta-analysis results.

**Fig. 4:**
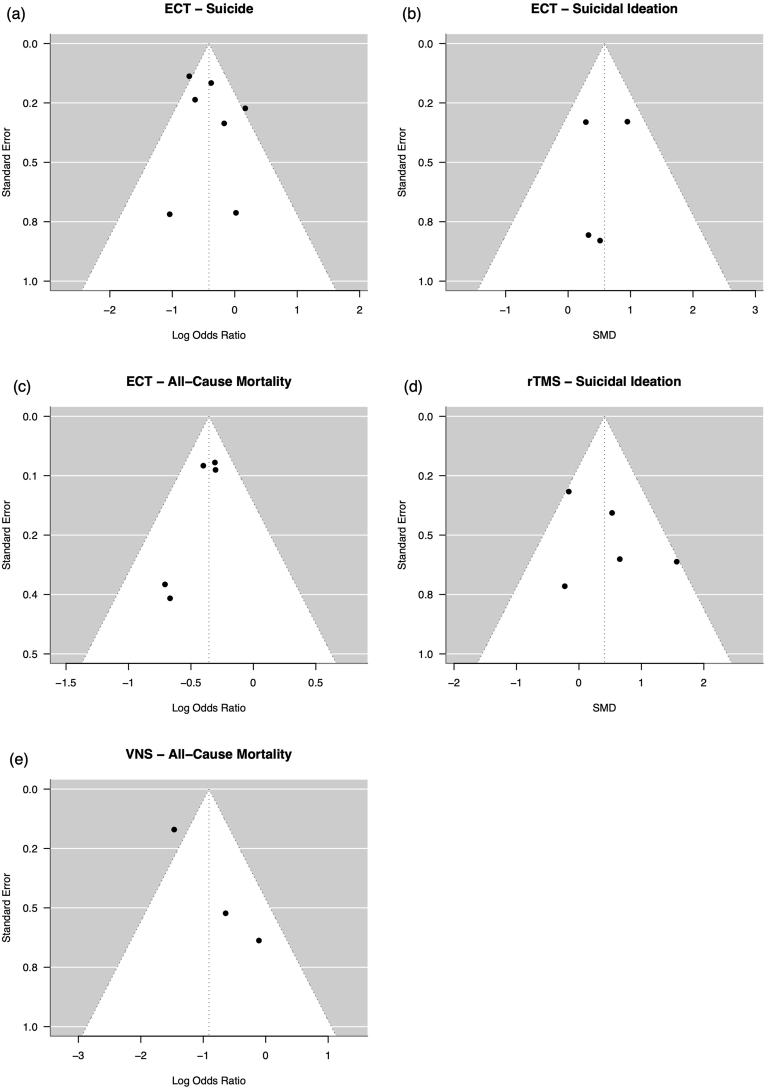
Funnel plot showing the estimate spread of the studies included in the meta-analysis.

A meta-analysis comprising a total of k = 7 studies (12′210 subjects treated with ECT and 19′671 subjects in the control group) was conducted to evaluate the comparative efficacy of ECT versus TAU. Follow-up durations and reporting methods varied: studies with range data had a minimum follow-up of 12 months, while those reporting medians had a minimum of 2.4 years (ECT) and 1.8 years (control), with a maximum median of 8 years. In the ECT group 208 suicide deaths (1.70 %) and in the control group 988 suicide deaths (5.02 %) were registered. Treatment with ECT demonstrated a significant 34 % decrease in the odds of suicide over the aggregated follow-up period (OR 0.66, 95 % CI 0.50–0.88, p = 0.0047; Fig. 3a). The heterogeneity of ORs across studies was quantified as medium with an *I*^*2*^ statistic of 51.7 %, although this did not reach statistical significance (p = 0.0698). No studies exerted disproportionate influence on the overall findings. Furthermore, Egger's test indicated no discernible asymmetry in the funnel plot (p = 0.4231; Fig. 4a). There was no evidence of excess significance bias (p = 0.3893). Despite the consistent results and low risk of bias, the observational nature of the majority of studies and the presence of some imprecision led us to assign a certainty of evidence rating of "very low." The credibility of the evidence was rated as "weak."

#### 3.2.2 ECT and suicidal ideation

The meta-analysis included k = 4 comparisons (204 subjects treated with ECT and 220 subjects receiving TAU) drawn from 3 separate publications. The derived average SMD was −0.58 (95 % CI –0.10 to −1.07, p = 0.0177; Fig. 3b), suggesting a moderate effect size of ECT on reducing suicidal ideation. Heterogeneity among the studies was minimal, indicated by an *I*^*2*^ value of 14.2 % and insignificance (p = 0.5399). None of the studies were found to exert unequal influence based on Cook's distances. We found no evidence of excess significance (p = 0.5468). Furthermore, there was no discernible asymmetry or small study effects (p = 0.7776; Fig. 4b). The presence of imprecision led us to assign a certainty of evidence rating of "moderate." The credibility was classed as “weak.”

#### 3.2.3 ECT and all-cause mortality

A total of k = 5 studies (the ECT group consisted of 16′332 subjects and the control group of 19′960 subjects) were included in this analysis. In the ECT group, there were 511 deaths (3.13 %), compared to 1325 deaths (6.64 %) in the control group. In the ECT group, a 30 % reduction in the odds of death from all causes was observed (OR 0.70, 95 % CI 0.62–0.79, p < 0.0001; Fig. 3c). There was no significant heterogeneity of the true outcome estimate (*I*^*2*^ 0 %, p = 0.6810), and none of the studies exerted excessive influence. The test for excess significance showed no evidence of bias (p = 0.2012). Additionally, there was no indication of funnel plot asymmetry (p = 0.0630; Fig. 4c). Moreover, the study published by Rhee et al. further supported ECT's protective effect against all-cause mortality (featuring >41,000 patients). However, it could not be included in the meta-analysis due to the unavailability of raw event data (Rhee et al., 2021). The certainty of evidence was graded “very low” due to reliance on observational data and presence of imprecision. Nevertheless, the credibility was “convincing” (Class I).

### 3.3 rTMS and suicidal ideation

Seven studies investigated the relationship between rTMS (including TBS/iTBS) and suicidal thoughts. Two of them also collected data on suicide deaths and all-cause mortality but no statistical analysis was possible because zero events were reported during the study period. The meta-analysis included 5 studies involving 197 patients treated with rTMS and 206 patients with TAU. Follow-up time ranged from at least 7 days up to at most 6 months. Suicidal ideation was measured by the Beck Scale of Suicidal Ideation in four out of five studies and in one study by the self-rating idea of suicide scale (SIOSS). All of them were randomized, sham-controlled studies. One study (Desmyter et al., 2016) used a cross over-design where we only included the results of the first phase (before they switched) to avoid a carryover-effect. We had to exclude one study (George et al., 2014) because of missing data on standard deviations of the measured data. We excluded one study due to its identification as a predecessor. The subsequent follow-up study expanded upon and included the cohort from this predecessor (Pan et al., 2020, 2023).

The meta-analysis incorporated 5 studies. The calculated SMD was statistically insignificant at −0.41 (95 % CI –1.01 – 0.19, p = 0.1795; Fig. 3d), indicating no significant effect of rTMS on suicidal ideation. There was no substantial heterogeneity in the true outcomes (*I*^*2*^ 46.9 %, p = 0.1067), and none of the studies were deemed overly influential. No evidence of excess significance was detected (p = 0.5641). Furthermore, no funnel plot asymmetry was detected (p = 0.3655; Fig. 4d). The certainty of evidence was graded “moderate.” The credibility was considered “no evidence” (Class V).

### 3.4 VNS and all-cause mortality

Two prospective observational studies and one retrospective comparative study examined the associations between VNS and all-cause mortality. Follow-up time varied from 2 up to 9 years. Studies consisted of 1′519 subjects treated with VNS and 12′756 controls. In the group treated with VNS 49 deaths (3.23 %) were registered versus 2409 deaths (18.87 %) in the control group. No studies reporting on VNS and suicide rates or suicidal ideation were identified.

In the VNS arm, a 60 % decrease in the odds of death from all causes was noted (OR 0.40, 95 % CI 0.18–0.92, p = 0.0306; Fig. 3e). There was a statistically significant moderate heterogeneity of the true outcome estimate (*I*^*2*^ 64.9 %, p = 0.0494). Based on Cook's distances, none of the studies were deemed overly influential. No excess significance was observed (p = 0.7742). The Egger's test showed no significant funnel plot asymmetry (p = 0.0730; Fig. 4e). We graded the certainty of evidence “very low” due to imprecision. The credibility was “weak” (Class V).

## 4 Discussion

### 4.1 Principal findings

Despite numerous efficacy trials assessing biological agents tailored to DSM diagnoses, there remains a scarcity of sufficiently powered RCTs investigating the effectiveness of biological interventions in mitigating suicide deaths, attempts, and ideation as distinct outcomes (O'Neil et al., 2012; O'Connor et al., 2013). To our knowledge, this represents the first meta-analysis demonstrating a survival benefit for ECT in depressive disorders. Our meta-analysis shows positive results for ECT in terms of reducing suicide deaths and all-cause mortality. A key observation is the trend of more pronounced positive effects in newer studies, particularly those published since 2016, which are generally of higher quality, characterized by larger sample sizes, prospective designs, and rigorous matching. The improvement in study quality corresponds with better outcomes, providing further evidence of a genuine protective effect. Another factor contributing to the observed trend of heightened protective efficacy against suicide may stem from the progressive improvement and standardization of ECT techniques and protocols. This evolution ensures uniformity in procedure execution, thereby promoting enhanced efficacy and favourable patient outcomes (Tirmizi et al., 2012). Previously, Wilkinson and colleagues did not ascertain a statistically significant effect of ECT (Wilkinson et al., 2022). We attribute this discrepancy to two primary limitations. Firstly, newer, well designed studies published subsequent to 2018 have not been incorporated into that review (Kaster et al., 2022; Rönnqvist et al., 2021). Secondly, the heterogeneity introduced by including patients with varied diagnoses, encompassing mood disorders, psychotic disorders, and post-traumatic stress disorder (PTSD), might serve as a constraining factor. Consequently, we intentionally restricted our analysis to depressive disorders as the main diagnosis, as neurostimulation in this domain has been extensively investigated and validated. Moreover, disparate factors may underlie suicidal tendencies across different psychiatric diagnoses (Maung, 2022). Similarly, Kucuker et al. deduced from their systematic review that ECT exerts a favourable influence on suicide reduction. However, this conclusion is less dependable due to the inclusion of heterogeneous diagnoses and varying study designs, including uncontrolled studies. The heterogeneity precluded a meta-analytic quantitative synthesis of effect size and certainty estimates (Kucuker et al., 2021). ECT is thought to alleviate depression through multiple mechanisms, and while its suicide-protective effect is likely driven by its antidepressant efficacy, limited post-mortem and rodent studies suggest a possible direct anti-suicidal effect, though this remains uncertain (Singh and Kar, 2017; Weitz et al., 2014; Lillethorup et al., 2015).

We observed a moderate reduction in suicidal ideation with ECT, as assessed by standardized tools in studies focusing on this outcome. However, such an effect could not be replicated with rTMS. Nonetheless, comparing these treatments is challenging, as unlike ECT studies, data on rTMS primarily originates from RCTs. Herein lies the limitation of RCTs, as these trials are typically constrained by high expense, resulting in smaller sample sizes, shorter durations, and often involve selecting patients with fewer risk factors associated with a complex course, who typically exhibit a higher suicide risk (Tondo and Baldessarini, 2024; Lawrence et al., 2024). As the FDA initially approved rTMS in 2008 (Horvath et al., 2011), which was 70 years following the inaugural utilization of ECT in modern medicine, rTMS has not had adequate time to become widely available. Consequently, reliable outcome data from large study populations over extended periods of time are still being gathered. Therefore, it is crucial to emphasize that the current lack of evidence for a suicide protective effect should not be interpreted as evidence of absence. Furthermore, it must be considered that a decrease in suicide rates and a decrease in suicidal ideation or thoughts should not be equated. The direct impact of reductions in suicidal ideation on suicide mortality remains uncertain (Griffiths et al., 2023). Hence, it cannot be assured that it serves as a suitable "surrogate endpoint" for the "hard endpoint" of suicide death. A critical limitation in studying the effects of rTMS on suicidality is that, in routine clinical practice, suicidality is often considered a contraindication for its use, as reflected in certain clinical guidelines (Tikka et al., 2023). This restricts the availability of reliable registry-based observational data from real-world clinical practice.

Patients with psychiatric conditions spanning various mental disorders are considered a high-risk demographic with elevated all-cause mortality beyond that of suicide mortality, attributable to diverse factors (Walker et al., 2015). The meta-analysis revealed that depression was linked to a 52 % heightened risk of all-cause mortality (Cuijpers et al., 2014). In comparison to other individuals with depression, patients struggling with refractory depression are at an even higher risk for all-cause mortality (Li et al., 2019). In the realm of general medicine, mortality endpoints for treatments like cardiovascular interventions have been standard practice since the mid-1980s (ISI S-2 (SECOND INTERNATIONAL STUDY OF INFARCT SURVIVAL) COLLABORATIVE GROUP, 1988). However, mortality endpoints are underemphasized in the psychiatric literature (Ahrens, 1994; Bushe et al., 2010). Ahrens contends that psychiatric researchers should prioritize normalized mortality as one of the most reliable indicators of treatment efficacy in studies assessing psychiatric treatment effectiveness (Ahrens, 1994). Regarding all-cause mortality in mood disorders, the efficacy of psychopharmacology presents a heterogeneous landscape. On one hand, lithium has consistently exhibited a reduction in all-cause mortality among patients with bipolar disorder (Toffol et al., 2015; Chen et al., 2023). Conversely, a protective effect of antidepressants against suicide and all-cause mortality remains a subject of ongoing debate (Soumerai et al., 2024). Concerningly, studies indicate an increase in mortality risk associated with long-term antidepressant use in the general population, particularly due to elevated cardiovascular event risks (Hansen et al., 2016; Maslej et al., 2017). Psychotropic polypharmacy is prevalent among individuals with difficult-to-treat depression (Rhee and Rosenheck, 2019), a practice that remains controversial due to the associated risk of increased mortality (Leelakanok et al., 2017). ECT often enables reduction in the number and dose of psychopharmacological agents (Zöllner et al., 2020) and might thus decrease direct and indirect serious adverse medication events. Another intriguing hypothesis would suggest that improved depression treatment outcomes may longitudinally correlate with better lifestyle choices and self-care practices. However, unfortunately, there are currently no studies investigating these aspects of ECT outcomes. Our findings are further supported by a large, nationally representative cohort study of Medicare-insured psychiatric inpatients aged 65 and older, which examined the effects of ECT on suicide and all-cause mortality risk (Rhee et al., 2021). While the anti-suicidal effect observed in this cohort was short-lived, the substantial reduction in all-cause mortality suggests that the survival benefit of ECT extends beyond suicide prevention alone. Notably, patients receiving ECT had a lower all-cause mortality risk for up to one year following hospital discharge compared to the control group. This is particularly compelling given the advanced age and multiple comorbidities of the study population, indicating that the therapeutic effects of ECT may extend beyond psychiatric symptom relief to broader improvements in overall health. The study's use of exact matching and rigorous confounder adjustment further enhances its reliability (Rhee et al., 2021). In summary, the observation of a 30 % reduction in all-cause mortality likelihood supports the long-term efficacy of ECT in depressive disorder. It could also be interpreted as another indicator of the long-term safety of ECT.

Regarding VNS and all-cause mortality reduction, there are insufficient studies, and the favourable outcome could be primarily driven by the study of Feldman et al. (2013), even though the formal test did not indicate this. However, the authors were unable to specify a cause for this difference in mortality due to a lack of data on causes of death and factors associated with mortality (Feldman et al., 2013). Despite the sound methodology employed in this study, it is important to acknowledge that industry sponsorship poses a limitation. Nonetheless, the data is encouraging, suggesting that VNS could offer a salvaging option in refractory serious mental illnesses where other treatments have proven ineffective. This underscores the need for further investigation in the future. However, the duration until a therapeutic effect is noticeable is very long for VNS (months), rendering it a more long-term treatment alternative with regard to suicidality and mortality (Jung et al., 2023).

#### 4.1.1 Strengths and limitations of study

The findings from a meta-analysis are inherently limited by the quality and limitations of the original data. Suicide research in psychiatry is constrained by several well-recognized general limitations. Individuals with suicidal ideation and a history of suicide attempts have conventionally been excluded from investigations into biological treatments, due to scientific and ethical considerations. However, individuals with a history of prior suicide attempts face a significantly elevated risk of completed suicide, ranging from 40 to over 100 times higher than that of the general population (Hawton et al., 2003; Runeson, 2002). Therefore, suicide protective interventions should be targeted towards these individuals. The primary evidence regarding biological interventions in suicide prevention largely stems from post hoc analyses (National Collaborating Centre for Mental Health (UK), 2012). Additional challenges in studying the impact of biological treatments on suicide, as well as their potential risks and protective effects, include systematic underreporting, questionable representativeness of patient samples, media-driven reporting, duplication of reports, and extensive missing information (Health Affairs Health Policy Brief, 2015). Conducting direct investigations on patients at high risk for suicide, with specific focus on acute precipitating factors and opportunities for intervention, presents inherent challenges. The populations are particularly vulnerable, and events are rare yet life-threatening, so testing singular interventions in the manner expected in high-quality biomedical studies is particularly challenging. Ensuring safety while simultaneously monitoring and controlling for therapeutic variables other than the purported suicide risk-mitigating treatment poses considerable complexity (Griffiths et al., 2014). Numerous psychological vulnerabilities predisposing individuals to suicide risk are well established, encompassing factors such as hopelessness, low self-esteem, impulsivity, impaired problem-solving abilities, maladaptive decision-making, deficient reality testing, and cognitive inflexibility (Richard-Devantoy et al., 2014). However, the neurobiological underpinnings of these vulnerabilities and their associated constructs remain poorly understood. Consequently, elucidating how proposed biological agents may alleviate them at a neurophysiological level poses a challenge (Griffiths et al., 2014). There is a paucity of studies systematically investigating the mechanisms through which biological agents could specifically influence suicidal behaviour (Gibbons et al., 2019). Enhanced comprehension of neurobiology in the future may facilitate the development of more effective intervention tools to alleviate suicidality. Studies designed to elucidate the interface between biology and various environmental factors that influence suicide risk may lead to the development of more sophisticated interventions. Importantly, our systematic review and meta-analysis focusing on neurostimulation does not exclude the possibility of other interventions being effective in cases of severe or refractory depressive states. Various forms of psychotherapy and other promising psychosocial interventions play crucial roles in suicide prevention (Mann et al., 2021; Gysin-Maillart et al., 2016), but they are outside the scope of this review and are not addressed here. Our findings assume greater significance considering the current status of other biological agents purported to possess potential anti-suicidal effects in individuals with mood disorders. Specifically, lithium and ketamine, both under discussion, have encountered scrutiny. Suicidal events are too infrequent in lithium trials to draw definitive conclusions (Tondo and Baldessarini, 2024; Nabi et al., 2022), while a recent meta-analysis suggests that ketamine's efficacy in depressive states could potentially be attributable solely to placebo effects (Matsingos et al., 2024).

One limitation in the included studies is the variability in the availability of potentially pertinent clinical details, particularly the lack of detailed information on treatments within the TAU groups. This variability is anticipated due to the typically prolonged and intricate course of refractory depression, leading to a complex and heterogeneous array of treatments. Despite frequent discussion, there remains a significant lack of research on the influence of expectation and placebo/nocebo effects on ECT outcomes. However, existing evidence fails to substantiate a significant impact of these factors within the context of refractory and chronic depression (Brodaty et al., 2003; Krech et al., 2018). A critical limitation is that many ECT studies are retrospective and/or non-randomized which may lead to biased results. However, due to the established efficacy of ECT in past RCTs and the critical condition of patients who are usually receiving ECT for ethical reasons (Espinoza Randall and Kellner Charles, 2022), it is unlikely that long-term RCTs on ECT for depression will be conducted in the near future. Predictably, the retrospective observational design of most primary studies reporting suicide and mortality outcomes has limited the certainty and credibility of our analyses. Given these constraints, substantial improvements in evidence quality are unlikely in the foreseeable future. In psychiatric RCTs on depression, it is well recognized that there is a bias toward enrolling patients with mild to moderate depression, often excluding those with DTD (Mulder et al., 2018). However, quite the opposite applies to rTMS and particularly ECT (Kiebs et al., 2019). ECT is typically reserved for severe, “treatment-resistant” cases, where despite its efficacy response rates are still lower than in non-TRD populations (Hsieh et al., 2023). A recent nationwide cohort study reported response rates of 65.9 % in the TRD group compared to 75.9 % in the non-TRD group (Nygren et al., 2023). This finding suggests a potential negative publication bias, which may lead to an underestimation of both the antidepressant and potentially anti-suicidal effects of ECT and rTMS. We relied on unadjusted effect estimates due to the variability in covariate adjustment across studies. The absence of individual participant data prevented us from providing consistently adjusted effect estimates (Riley et al., 2010). The studies included in the analysis predominantly originate from high-income developed countries. Further research is needed to enhance our understanding of outcomes in other regions.

A critical domain for future inquiry pertains to enhancing access to therapies demonstrated to mitigate the risk of suicidal behaviours, as well as refining the alignment of appropriate therapies with specific indications. In the future, adequately powered, multi-centre studies utilizing patient registries are imperative to gather larger-scale data for assessing treatment effects and monitoring long-term outcomes. There is growing advocacy within the field for enhanced standardization of methodology and outcome measures (e.g., suicidal ideation, suicidal behaviour, suicidal mortality) and the creation of large multicentric databases to enhance the longevity and comparability of data collected across multiple smaller studies (Insel et al., 2011).

## Declaration of competing interest

The authors declare that they have no conflicts of interest to disclose related to the present manuscript or the treatment techniques described herein.
