# Supplementary material for: Electroconvulsive therapy reduces suicidality and all-cause mortality in refractory depression: A systematic review and meta-analysis of neurostimulation studies
Source: Neurosci Appl. 2025 Jun 2;4:105520. doi: 10.1016/j.nsa.2025.105520 (PMC12664644; doi:10.1016/j.nsa.2025.105520)
Supplement: Multimedia component 1 [file mmc1.docx]

Supplementary Table 1. Quality Assessment Using the GRADE Framework of Each Pooled Analysis

| **GRADE Criteria** | **Assessment** | **Action** |
| --- | --- | --- |
| ECT – Suicide | | |
| *Domains lowering certainty* | | |
| Starting Level | Observational evidence | Begins at "low" |
| Risk of Bias | Not serious | No downgrading |
| Inconsistency | Not serious | No downgrading |
| Indirectness | Not serious | No downgrading |
| Imprecision | Present | 1 downgrade |
| Publication Bias | None | No downgrading |
| *Factors potentially increasing certainty not applicable because the limitation of imprecision is present* | | |
| Final Certainty of Evidence: Very Low | | |
| ECT – Suicidal Ideation | | |
| *Domains lowering certainty* | | |
| Starting Level | All studies are controlled clinical trials. | Begins at “high” |
| Risk of Bias | Not serious | No downgrading |
| Inconsistency | Not serious | No downgrading |
| Indirectness | Not serious | No downgrading |
| Imprecision | Present | 1 downgrade |
| Publication Bias | None | No downgrading |
| Final Certainty of Evidence: Moderate | | |
| ECT – All-cause Mortality | | |
| Starting Level | Observational evidence | Begins at "low" |
| Risk of Bias | Not serious | No downgrading |
| Inconsistency | Not serious | No downgrading |
| Indirectness | Not serious | No downgrading |
| Imprecision | Present | 1 downgrade |
| Publication Bias | None | No downgrading |
| Final Certainty of Evidence: Very Low | | |
| *Factors potentially increasing certainty not applicable because the limitation of imprecision is present* | | |
| rTMS – Suicidal Ideation | | |
| *Domains lowering certainty* | | |
| Starting Level | All studies are controlled clinical trials. | Begins at “high” |
| Risk of Bias | Not serious | No downgrading |
| Inconsistency | Not serious | No downgrading |
| Indirectness | Not serious | No downgrading |
| Imprecision | Present | 1 downgrade |
| Publication Bias | None | No downgrading |
| Final Certainty of Evidence: Moderate | | |
| VNS – All-cause Mortality | | |
| Starting Level | Observational evidence | Begins at "low" |
| Risk of Bias | Not serious | No downgrading |
| Inconsistency | Not serious | No downgrading |
| Indirectness | Not serious | No downgrading |
| Imprecision | Present | 1 downgrade |
| Publication Bias | None | No downgrading |
| Final Certainty of Evidence: Very Low | | |
| *Factors potentially increasing certainty not applicable because the limitation of imprecision is present* | | |
