# Supplementary material for: Electroconvulsive therapy reduces suicidality and all-cause mortality in refractory depression: A systematic review and meta-analysis of neurostimulation studies
Source: Neurosci Appl. 2025 Jun 2;4:105520. doi: 10.1016/j.nsa.2025.105520 (PMC12664644; doi:10.1016/j.nsa.2025.105520)
Supplement: Multimedia component 2 [file mmc2.docx]

Supplementary Table 2. Credibility of evidence assessment criteria

| **Classification** | **Criteria** |
| --- | --- |
| Convincing (Class I) | - Number of cases > 1000 (or > 20,000 for continuous outcomes)  - P<1×10−6  - I^2^<50%  - 95% prediction interval excludes null hypothesis  - Largest included individual study exhibits P≤0.05  - No small-study effects  - No excess significance bias |
| Highly Suggestive (Class II) | - Class I criteria not all met  - Number of cases > 1000  - P<1×10−6  - Largest included individual study exhibits P≤0.05 |
| Suggestive (Class III) | - Class I—II criteria not all met  - Number of cases > 1000  - P<1×10−3 |
| Weak (Class IV) | - Class I—III criteria not all met  - P≤0.05 |
| No evidence (Class V) | - P>0.05 |
