# Supplementary material for: Electroconvulsive therapy reduces suicidality and all-cause mortality in refractory depression: A systematic review and meta-analysis of neurostimulation studies
Source: Neurosci Appl. 2025 Jun 2;4:105520. doi: 10.1016/j.nsa.2025.105520 (PMC12664644; doi:10.1016/j.nsa.2025.105520)
Supplement: Multimedia component 3 [file mmc3.docx]

Supplementary Table 3: Descriptive analysis of all studies included in systematic review

| **Author, year, country** | **Study aim** | **Study design** | **Follow-up time (years of data collection)** | **Study population** | **Assessment tools** | **Treatment modality** | **Treatment procedure** | **Outcome** | **Quality of evidence^a^** | **Overall Risk of Bias^b^** |
| --- | --- | --- | --- | --- | --- | --- | --- | --- | --- | --- |
| **ECT** | | | | | | | | | |  |
| Avery et Winokur (1976), USA | To compare ECT, antidepressant therapy or neither treatment for suicide and total mortality. | Comparative study | 3 years | 519 hospitalized patients with a DSM I or II depressive diagnosis, 62% women, mean age 51.5 years. | Follow-up letters, death certificates by the Health Department of the State of Iowa. | 257 subjects received ECT, 262 subjects received no ECT. | 7 subjects received less than 5 treatments, 140 subjects 5-9 treatments, 74 subjects 9-11 treatments, 36 subjects 12 or more treatments. | Suicide and total mortality | Good (4/2/3) | Moderate |
| Babigian et Guttmacher (1984), USA | To find out how rates of use of ECT have changed between 1961-1975, who has received ECT, if there was a relationship between use of ECT and length of hospitalization and if there was an altered risk of mortality associated with ECT. | Comparative study | 5 years (1961-1980) | 3927.9 patients with first lifetime psychiatric hospitalizations with endogenous depression or neurotic depression, 68% women, 63% were older than 45 years. | Monroe County (New York) Psychiatric Case Register, Mortality information available by the New York State Department of Health. | 818 subjects received ECT, 3109 subjects received no ECT. | No information. | Suicide and total mortality | Good (4/2/3) | Low |
| Brådvik et Berglund (2000), Sweden | To identify any difference in treatment between depressed patients wo have contributed to the suicidal outcome. The treatment at last contact before suicide was measured. | Retrospective case-control study | 9.5 ± 9.2 years in the suicides group and 8.1 ± 9.1 years in the control group (1956-1984) | 178 patients, 91% of the patients met the criteria for a major depressive disorder with melancholic, psychotic or catatonic features. The mean age at last contact was 50.5 years in the suicide group and 49.3 years in the control group. | Case records from St. Lars Hospital, Lund, Sweden and from the psychiatric clinic and mental hospital in Helsingborg, Sweden. | 49 patients received ECT, 129 received no ECT (antidepressant therapy, inadequate therapy with ECT or antidepressant, lithium, untreated). | At least six treatments (given three times a week). Conventional ECT equipment (Siemens Convulsator 622; Erlangen, Germany) and procedures were used. The electrode placement was bifrontotemporal, except in two cases of unilateral nondominant placement in the 1970s (after 1971). The electric stimulus was manually regulated during each treatment to achieve an adequate grand mal seizure (> 25 seconds), usually 0.6 to 0.9 ampere. The voltage was 180 V. The currency was unidirectional. At the time, no psychotropic medication was given during ECT. | Differences in treatment between depressed patients who committed suicide and matched patients who did not that might have contributed to the suicidal outcome. | Good (4/2/2) | Critical |
| Nordenskjöld et al. (2013), Sweden | To test the hypothesis that relapse prevention with continuation electroconvulsive therapy (ECT) plus pharmacotherapy is more effective than pharmacotherapy alone after a course of ECT for depression. | Multicenter, nonblinded, randomized controlled trial with 2 parallel groups | 12 months (01/2008-03/2012) | 56 patients with mayor depression (single episode, recurrent or bipolar) who received ECT treatment (no more than 3 weeks since last ECT). In the ECT group 57% were female and the mean age was 52 years. In the control group 43% were female and the mean age was 62 years. | Patients of 4 hospitals in the middle of Sweden. | 28 patients treated with continuation ECT and pharmacotherapy. 28 patients treated with pharmacotherapy alone. All patients received individualized medication. The pharmacotherapy at randomization consisted of antidepressants (98%), lithium (56%), and antipsychotics (30%). | Weekly ECT for 6 weeks and thereafter every 2 weeks for 46 additional weeks, a total of 29 ects for the full year. Unilateral ultrabrief pulse ECT was used. At the first continuation ECT session, the seizure threshold was measured and at the proceeding sessions, an electrical dose 6 times the seizure threshold was used. The Mecta Spectrum 5000Q device (Mecta Corp, Lake Oswego, Ore) was used at 3 hospitals and a Thymatron System IV (Somatics, Inc, Lake Bluff, Ill) was used at one hospital. The mean (SD) electrical dosage during continuation ECT was 0.36 (0.11) milliseconds (ms), 74 (20) Hz, 6.40 (1.21) seconds, 813 (35) ma, and 292 (166) mc. At least 20-second electroencephalogram recorded epileptic activity was sought. | Relapse of depression within 1 year (Relapse was defined as 20 points or more at interview-based MADRS, or inpatient-psychiatric care for any reason, or suicide, or suspected suicide). | - | Some concerns |
| Ahmadi et al. (2016), USA | To investigate the efficacy of ECT on long-term clinical outcome of comorbid PTSD and MDD. | Retrospective cohort study, using a nested matched case-control design | Mean follow up of 8 years (2004-2013) | 3485 patients with both major depressive disorder (MDD) and posttraumatic stress disorder (PTSD) with age, gender and risk factors matched. In the ECT group 85% were male and the mean age was 52 ± 12 years. In the control group 86% were male and the mean age was 53 ± 10 years. | VHA administrative, research, and clinical electronic medical records, Social Security Death Index obtained from electronic medical records including VA Beneficiary Identification and Records Locator system (BIRLS), VA Centers for Medicare & Medicaid, Services (CMS) vital status, Social Security Administration death (SSA), National Death Index Data. | 92 patients treated with ECT and 3393 patients without ECT treatment. They were all on antidepressant therapy. | ECT was performed using a Thymatron IV ECT device (Somatics LLC, Lake Bluff, Illinois, USA). All patients received bifrontal (BF) ECT, with stimulus dose administration at least 200% above the seizure threshold based on age-based method, three times a week with an average of 6 ± 1 ECT sessions. | All-cause-mortality and suicide | Good (4/2/3) | Moderate |
| Liang et al. (2018), Taiwan | To investigate whether ECT outperforms psychopharmacotherapy in reducing suicidal intent in patients with affective disorders. | Nationwide retrospective cohort study | The mean follow-up time was 4.91 ± 4.28 years for the ECT group and 4.05 ± 4.12 years for the non-ECT group (01/2000-12/2013). | Patients with unipolar or bipolar disorders. In the ECT group 35.7% were male and the mean age was 38.5 ± 14.2, in the control group 35.7% were male and the mean age was 39.2 ± 14.2. | Data collected from the Taiwan National Health Insurance Research Database (NHIRD), which are derived from the claims data of the National Health Insurance (NHI) program. | 487 patients treated with ECT and 1948 patients received psychopharmacotherapy. | No information. | Death by suicide | Good (4/2/3) | Low |
| Jørgensen et al. (2020), Denmark | To examine whether depression severity predicts the use of electroconvulsive therapy, risk of re-hospitalization, suicidal behaviour and mortality following electroconvulsive therapy in patients with major depression. | Nationwide retrospective cohort study | Unclear (01/2005-10/2016) | Citizens in Denmark with a first-time hospital contact due to single episode or recurrent depression. 38% men, well balanced for age, educational status and marital status. | Danish National Patient Registry (DNPR), Causes of Death Registry. | 5004 patients treated with ECT, 87891 patients not treated with ECT. | No information. | Rehospitalization with major depression as main diagnosis as inpatient or emergency ward patient, suicide attempts, suicide and all-cause mortality | Good (4/2/2) | Low |
| Kheirabadi et al. (2020), Iran | To compare the antidepressant and antisuicidal effects of oral and intramuscular (IM) ketamine versus electroconvulsive therapy (ECT). | Randomized study | 1 month | 45 patients with major depressive disorder. In the ECT group 33.3% were male and the mean age was 41.6 ± 15.44 years. In the oral ketamine group 40% were male and the mean age was 39.13 ± 9.84 years. In the i.m. Ketamine group 53.3% were male and the mean age was 41.6 ± 8.43 years. | Questionnaires. | 12 patients received ECT, 12 patients received oral ketamine, 15 patients received ketamine i.m. | 6 to 9 sessions of ECT for 3 weeks. The ECT electrical stimulus was firstly set at 20 J using DGX machine and then was titrated based on the duration of induced seizure. The ECT electrodes were placed bifrontotemporal. The duration of induced tonic-colonic seizure was considered to be at least 20 seconds to provide a suitable response. | Hamilton Depression Rating Scale and the Beck Scale for Suicidal Ideation scores | - | Some concerns |
| Lin et al. (2020), Taiwan | To compare existing data from 2 previously published open-label studies for MDD patients treated with ECT or fluoxetine. | Two open-label trials, non-blinded, non-randomized | 12 weeks (01/2008-10/2013 for the first study, 05/2007-02/2010 for the second study) | ECT group (first study): MDD patients, 80% were female, the mean age was 46.5 years.  Fluoxetine group (second study): MDD patients, without a history of treatment-resistant depression, 89% were female and the mean age was 45.6 years. | Two trials were conducted at the Psychosomatic Ward and Outpatient Department of the Kai-Syuan Psychiatric Hospital, Kaohsiung, Taiwan. | 111 subjects in the ECT group, 114 sibjects in the fluoxetine group (20 mg/d as a monotherapy for a period of up to six weeks). | ECT was conducted using the Thymatron System IV machine with brief pulse and constant current (pulse width, 0.5ms; frequency, 60 Hz; current, 0.9A). The initial stimulus dose was determined by an age-based, gender-adjusted method. Seizure duration was at least 20 seconds as measured by electromyogram, and 25 seconds as measured by electroencephalography. Treatment was given 3 times a week before August 2009, and later 2 times a week, with a maximum of 12 treatments. | Reduction of suicidal ideation, speed of resolution of suicidal ideation in inpatient treatment, time to relapse of suicidal ideation | Good (3/2/2) | Moderate |
| Rönnqvist et al. (2021), Sweden | To determine the association between ECT and the risk of suicide in patients with unipolar major depressive disorder. | Registry-based cohort study | 12 months (01/2012-10/2018) | Patients who had a record of inpatient care for moderate depression, severe depression or severe depression with psychosis were included. In the ECT group the mean age was 55.9 ± 18.4 years, in the non-ECT group it was 45.2 ± 19.2 years. 55.5% were female. | The Swedish National Inpatient Register, the Swedish National Quality Register for ECT, Swedish Prescribed Drug Register, Swedish Causes of Death Register, Longitudinal Integration Database for Health Insurance and Labour Market Studies and the Multi Generation Register. | 5525 patients in the ECT group and 5525 patients in the non-ECT group. | ECT was usually administered 3 times per week using the bidirectional constant-current brief-pulse Mecta (Mecta Corp) or Thymatron (Somatics Inc) device. The electrode application during ECT was unilateral in 86.5%, bilateral in 9.8%, and not known in 3.7%. | Suicide, all-cause mortality | Good (4/2/3) | Low |
| Kaster et al. (2021), Canada | To compare the risk of serious medical events, defined as those resulting in hospitalisation or death, among patients with depression who received electroconvulsive therapy versus patients who did not receive electroconvulsive therapy. | Propensity score-matched, retrospective cohort study | 30 days (04/2007-02/2017) | Adults (aged ≥18 years) with a discharge diagnosis of a major depressive episode during a psychiatric unit inpatient stay that lasted more than 3 days. In the ECT group the mean age was 56.4 ± 16.4 years and 66.2% were female. In the control group the mean age was 56.7 ± 16.2 years and 66.2% were female. Diagnoses were 81.1% unipolar depression, 15% bipolar depression. | Population-based administrative health-care databases at the ICES (formerly the Institute for Clinical Evaluative Sciences), Registered Persons Database, Canadian Institute for Health Information Discharge Abstract Database (CIHI-DAD), CIHI Ontario Mental Health Reporting System, CIHI National Ambulatory Care Reporting System, Ontario Health Insurance Plan Claims Database, Office of the Registrar General – Deaths. | 5008 patients in the ECT group and 5008 patients in the non-ECT group. | No information. | Hospitalisation, non-suicide death, suicide death and specific serious medical events | Good (4/2/2) | Low |
| Nordenskjöld et al. (2022), Sweden | To investigate the association between ECT and cardiovascular events within 1 year of admission to hospital for depression. | Nationwide, observational, registry-based cohort study | 1 year (01/2012-10/2018) | Patients admitted to Swedish hospitals due to moderate or severe unipolar. 33.8% were aged 18-44 years, 12.1% 45-54 years, 17.6% 55-64 years, 36.6% > 65 years. 43% were male. | Swedish National Patient Register, the Swedish Prescribed Drug Registry, the Swedish Cause of Death Register. | 5476 patients in the ECT group and 5476 patients in the non-ECT group. | No information. | Cardiovascular events, (stroke, myocardial infarction, cardiovascular death,) overall mortality | Good (4/2/3) | Low |
| Salagre et al. (2022), Denmark | To investigate the course of self-harm and suicide attempts preceding and following ECT across 4 major mental disorders. | Nationwide observational study | 2 years (1995-2019) | Patients with unipolar depression and bipolar disorder who received ECT for the first time.  Mean age ranged from 53.6-56.1 years, the number of men ranged from 36.2-38.8% in different diagnosis and treatment groups. | Data from the Danish Civil Registration System, the Danish National Patient Registry, and the Danish Register of Causes of Death. | 11'556 patients in the ECT group and 55'269 patients in the non-ECT group. | No information. | Intentional self-harm/suicide attempts, suicide | Good (4/2/3) | Low |
| Kaster et al. (2022), Canada | To compare the risk of death by suicide after psychiatric hospitalisation among individuals with depression who had been exposed to electroconvulsive therapy with those who had not. | Propensity score-weighted, retrospective cohort | 1 year (04/2007-12/2017) | Adults who had been discharged from a designated psychiatric inpatient bed in Ontario, for whom the primary diagnosis was a major depressive episode as part of a major depressive disorder or bipolar disorder. In the ECT group the mean age was 57.1 ± 16.8 years and 65.9% were female. In the control group the mean age was 44.2 ± 16.5 years and 59% were female. | 84 separate psychiatric inpatient units in Ontario, Canada. Registered Persons Database, Canadian Institute for Health Information Discharge Abstract Database (CIHI-DAD), CIHI Ontario Mental Health Reporting System, CIHI National Ambulatory Care Reporting System, Ontario Health Insurance Plan Claims Database, Office of the Registrar General – Deaths. | 4982 patients in the ECT group and 5304 patients in the unexposed group. | No information. | Death by suicide, non-suicide death, all-cause mortality | Good (4/2/3) | Low |
| Cai et al. (2023), China | To explore ECT effectiveness in improving suicidal ideation and depressive symptoms, as well as its impact on cognitive function in adolescents (aged 13-18 years) with major depressive disorder. | Nonrandomized controlled trial | 6 weeks (12/2017-09/2021) | Inpatients between 13-18 years old, diagnosed with MDD. In the ECT group the mean age was 15.25 ± 0.19 and 17.28% were male. In the control group the mean age was 14.87 ± 0.18 and 11.39% were male. | Patients were recruited at Beijing Huilongguan Hospital. Treatment response was assessed by experienced psychiatrists. | The ECT group (n = 81) group was treated with antidepressants and 8 rounds of ECT for 2 weeks. The control group comprised 79 patients treated with antidepressants only. | 8 rounds of ECT for 2 weeks. During the first week, ECT was applied daily, 5 times in succession; during the second week, ECT was applied 3 times every other day. Electrodes were placed on the bilateral temporal lobes, using a MECTA spectrum M5000Q (MECTA Corp, Tualatin, Oregon). The ECT parameters were as follows: maximum charge delivered, 504 mc; output current, 0.9 A; frequency, 10 to 70 Hz; pulse width, 1.0 ms; maximum stimulus duration, 8 seconds. | Suicide ideation, depressive symptoms (SIOSS and HAMD-17), cognitive function | Good (3/2/2) | Moderate |
| **TMS** | | | | | | | | | |  |
| George et al. (2014), USA | To test the safety, feasibility, and potential efficacy of delivering high doses of rTMS to suicidal inpatients. | Prospective, 2-site, randomized, active sham-controlled (1:1 randomization) | 6 months (12/2010-08/2013) | Inpatients that have been in a depressive episode (unipolar or bipolar II, non-psychotic) who were admitted because of suicidal ideation or attempt, aged 42.5 years +/- 15.7, 85% male. | This study was part of the injury and traumatic stress (intrust) Consortium, coordinated through the University of California. | 20 patients were allocated to active rTMS and 21 to sham rTMS. | We used a Model 2100 magnetic stimulator (Neuronetics, Inc., Malvern, PA; NS 0226 A 15VAC-C) consisting of a controller/power supply and three solid iron core coils (active labeled, active not labeled and sham not labeled) that were placed in a support platform for consistent coil position and repositioning. Repetitive TMS (rtms) was delivered to the left prefrontal cortex, defined as a location 6 cm(cm) anterior to the right hand motor thumb area. Rtmswas delivered with a figure-eight solid core coil at 120% motor threshold, 10 Hertz (Hz), 5 s (s) train duration, 10 s intertrain interval for 30 min (6000 pulses) 3 times daily for 3 days (total 9 sessions, 54,000 stimuli). | Primary outcomes were the daily change in severity of suicidal thinking as measured by the Beck Scale of Suicidal Ideation (SSI) administered at baseline and then daily (for three days) | - | Some concerns |
| Desmyter et al. (2016), Belgium | To examine the effects and safety of accelerated intermittent Theta Burst Stimulation (itbs) on suicide risk in a group of treatment-resistant unipolar depressed patients, using an extensive suicide assessment scale. | Randomized, sham-controlled cross-over study | 6 months | 50 therapy-resistant, antidepressant-free depressed patients. 35 were female, the mean age was 41.9 years. 32 subjects reported suicidal ideations at baseline (T1), which was defined by a BSI score of larger than 1. Consequently, all analyses were performed on these 32 suicidal subjects. | Suicide ideation was measured with the Beck Scale of Suicide Ideation (SSI) at four different time points: At baseline, after the first week of stimulation, after the second week of stimulation and 2 weeks after the last stimulation (so 1 month after baseline). Six months after baseline patients were contacted to assess whether or not they had committed suicide. | Patients were randomized to two groups: during the first week one group (n=14) received the active stimulation and the other group (n=18) started with the sham condition. They were switched to the other condition during the second week. | Intermittent TBS stimulation was applied using a Magstim Rapid Plus magnetic stimulator (Magstim Company Limited, Wales, UK) with a figure-of eight-shaped coil. A stimulation intensity of 110% of the patient’s resting motor threshold was administered during treatment. We used the Brainsight neuronavigation system (brainsight tm, rogue research, Inc.) To identify the site of stimulation based on a structural cerebral MRI of each individual in order to accurately target the left dorsolateral prefrontal cortex (DLPFC). iTBS was delivered at five sessions per day during 4 days. One iTBS session consisted of 54 trains of 10 bursts of three stimuli. These stimuli were applied in a 50 Hz frequency: the bursts were repeated every 200 ms. This resulted in 2 s of stimulation alternated by 8 s rest periods and 1620 stimuli per session. | Suicide ideation measured by the Beck Scale of Suicidal Ideation (SSI) | - | Low |
| Yesavage et al. (2018), USA | To determine the efficacy of rtms in the treatment of TRMD in veterans. | Double-blind, sham-controlled, randomized clinical trial | 24 weeks (09/2012-12/2016) | 164 veterans with treatment resistant major depressive disorder, the mean age was 55.2 years and 80.5% were men. Veterans with comorbid PTSD and a history of substance use disorders were not excluded. | Suicidal ideation was measured by the Beck Scale for Suicide Ideation and Columbia Suicide Severity Rating Scale. | 81 subjects received left prefrontal rtms treatment and 83 subjects received sham rtms treatment for up to 30 treatment sessions. | Repetitive transcranial magnetic stimulation was administered using a modified magpro R30 (magventure) device with Cool-B65-A/P coil. The A (active) side of the unmarked coil delivered active treatment and the P (placebo) delivered sham treatment. Approximately 4000 stimulation pulses/session, 10 Hz, 120% motor treshold. Participants received between 20 and 30 sessions of rtms. Participants who experienced remission after the initial 20 to 30 sessions received another 6 additional taper sessions that were delivered over a 3-week period. | Remission in depressive symptoms, suicidal ideation (measured by the Beck Scale for Suicide Ideation and Columbia Suicide Severity Rating Scale) | - | Low |
| Dai et al. (2020), China | To define the potential therapeutic effect of rtms to improve the clinical symptoms and reduce the suicidal ideation in elderly patients with depression. | Randomized, double-blind, parallel-group design study | 4 weeks (04/2015-04/2018) | 103 patients diagnosed with Depression, aged 60 to 80 years old, they had not taken any antidepressants within the last 3 months, typical clinical symptoms of depression, with HAMD score ≥20 and SIOSS score ≥12. The mean age was 68.2 ± 9.37 years, 65.1% of the subjects were female. | All cases were inpatients at the Department of Clinical Psychology of Jingmen NO.2 People’s Hospital. Suicide ideation was assessed by the SIOSS (self-rating idea of suicide scale). | 48 patients were allocated to active rtms and 55 to sham rtms. Both groups received routine drug therapy. | TMS device was manufactured by Magstim (United Kingdom). Figure of 8 coil and following treatment parameters were used based on preliminary experiments: stimulation frequency at 10 Hz, intensity of 100% motion threshold, duration of 20 minutes. Rtms was applied at left prefrontal lobe for 4 seconds per minute with an interval of 56 seconds (ie, 800 pulses per day) in 5 times per week for 4 weeks. | Hamilton depression scale and self-rating idea of suicide scale | - | Some concerns |
| Pan et al. (2020), China | To investigate the efficacy of MRI-based neuronavigation-guided daily high-dose rTMS for rapidly improving suicidal ideation in treatment-naive patients with major depressive disorder (MDD). | Double-blind sham-controlled study | 7 days | 42 treatment-naive patients between 13-45 years with current unipolar MDD with a score ≥ 20 on the 24-item Hamilton Depression Rating Scale (HAMD-24) and with a score ≥ 12 on the Beck Scale of Suicidal Ideation (BSI). Mean age in the active rtms group was 18.14 ± 3.94 and 21.43 ± 6.79 in the sham group. In the active rtms group 2/21 patients were male and in the control group 5/21. | The patients were recruited from the Department of Psychiatry at the First Affiliated Hospital, College of Medicine, Zhejiang University. | 21 patients received active rTMS and 21 patients received sham rTMS. The medication included escitalopram oxalate tablets (10 mg/d). The subjects started to take escitalopram oxalate tablets (10 mg/d) since the first day of active or sham rTMS treatment. | The rTMS treatment was delivered by a Magstim Rapid2 rTMS devices (The Magstim Company, Whitland, UK) with an eight-figured coil. We adopted rTMS treatment for 7 consecutive days over 1 week. Each daily rTMS session comprised 120 trains of 5 seconds duration at 10 Hz with inter-train intervals of 15 seconds (i.e., 6,000 pulses per session). The stimulus intensity was 100% of the resting motor threshold based on the tolerance of the participant. | Suicide ideation measured by the Beck Scale of Suicidal Ideation (SSI). Depression measured by the 24-item Hamilton Depression rating scale (HAMD) and Montgomery–Åsberg Depression Rating Scale (MADRS) | - | Some concerns |
| Pan et al. (2023), China | To investigate if rTMS improves suicidal ideation and depressive symptoms by influencing brain derived neurotrophic factor (BDNF), tropomysin receptor kinase B (trkb) and VGF levels. | Double-blind, randomized, sham-controlled study | 7 days | 59 treatment-naive depressive patients with suicidal ideation, with a score ≥ 20 on the 24-item Hamilton Depression Rating Scale (HAMD-24); and with a score ≥ 12 on the Beck Scale of Suicidal Ideation (BSI). The mean age in the active rTMS group was 18 ± 3.96 years and 20.60 ± 5.77 years in the sham group. In the active rTMS group 10 patients were male and in the control group 8 were male. | The patients were recruited from the Department of Psychiatry at the First Affiliated Hospital, College of Medicine, Zhejiang University. | 31 patients received active rTMS and 28 patients received sham rTMS. Each subject would take escitalopram oxalate tablets (10 mg/day). On the first day of active or sham rTMS treatment, each subject started to take medication. | The structural MRI-guided method was used to locate the stimulation target. MRI data of all subjects were acquired through the Signa hdxt 3.0 T MRI system (GE Healthcare, Milwaukee, WI) at baseline. The Magstim Rapid2 stimulator (The Magstim Company Ltd., Whitland, UK) with an eight-figured coil was used for treatment. We adopted the 10-Hz, 6000 pulses per session, 100 % of the MT parameter for rTMS treatment. Each patient underwent rTMS treatment for 7 consecutive days over one week. Each session included 6000 stimuli/day delivered in 120 trains of 5 s duration at 10 Hz with inter-train intervals of 15 s. | BDNF, trkb, VGF, BSI scores, HAMD scores, MADRS scores | - | Some concerns |
| Zhao et al. (2023), China | To examine the clinical efficacy of iTBS on reducing suicidal ideation in adolescent MDD with suicide attempt. | Randomized, single-blind, sham-controlled study | 2 weeks (03/2021-01/2022) | 45 patients with at least one attempted suicide within the last year, a score of 20 and higher on the Hamilton Rating Scale for Depression (HAMD-24) item and a score of 6 and higher on the Beck Scale for Suicide Ideation-Chinese Version (BSI-CV) item. Mean age was 17.20 ± 2.25 years, 17.8% male. | Patients were recruited from the inpatient ward of Chengdu Mental Health Center. | 23 patients received active iTBS, 22 received sham iTBS. | The iTBS sessions were administered with a figure-of-eight shaped coil by using an YRD CCY-I magnetic simulator (YIRUIDE Inc., Wuhan, China). The patients in the active iTBS group received a total of 10 sessions on the left dorsolateral prefrontal cortex (DLPFC) once a day on weekdays for two weeks. In each session, patients received 1800 iTBS pulses in 60 triplet bursts with a train duration of 2 s and an intertrain interval of 8 s. | Suicidal ideation and depressive symptoms using Beck Scale for Suicide Ideation-Chinese Version (BSI-CV), Hamilton Rating Scale for Depression (HAMD-24) and Self-rating Depression Scale (SDS) | - | Some concerns |
| **VNS** | | | | | | | | | |  |
| Olin et al. (2012), USA | To compare the performance of standard pharmacological and non-pharmacological therapies (TAU; including ECT and psychotherapy) to vagus nerve stimulation therapy (VNS+TAU) adjunctive to standard therapies. | Observational, prospective, open-label, longitudinal, multicenter study | Average follow-up of 3.2 years in the VNS+TAU group vs. 2.1 years in the TAU group. | Patients diagnosed with a current major depressive episode, mean age of 49.8 years for the TAU group and 48.8 for the VNS+TAU group, 70.1% female in the TAU group and 68.4% in the VNS+TAU group. | Treatment-Resistant Depression Registry; patients registries of 45 US centers. | 301 patients received standard treatment-as-usual (TAU) pharmacotherapy, where all available therapeutic interventions are allowed, including ECT and psychotherapy; 335 patients received VNS Therapy adjunctive to treatment-as-usual pharmacotherapy (VNS+TAU). | VNS Therapy consists of a small pulse generator surgically implanted in the chest that delivers intermittent stimulation (typically 30 seconds on, 5 minutes off) via an electrode partially wrapped around the left vagus nerve in the mid-cervical region; the electrical signals are in turn processed in the nucleus tractus solitarius and relayed to various regions of the brain. | All-cause mortality rate, suicide rate, rate of suicidal ideation | Good (4/2/3) | Low |
| Feldman et al. (2013), USA | To study the healthcare utilization experience of Medicare beneficiaries implanted with VNS (vnsbs) during Medicare coverage, compared with beneficiaries with TRD (trdbs) and managed depression (Mdeps). | Retrospective analysis | 8-10 quarter years (2001-2009).  About one-third of TRD patients and one-quarter of managed depressed patients were observed for 8–9 years | VNS population: All Medicare FFS beneficiaries who received VNS for depression and who did not have epilepsy. TRD sample: 5% sample of all Medicare FFS beneficiaries who in the 2-year identification period had major depression and averaged two or more psychiatric hospitalizations per year, averaged at least one medication management visit each 6 weeks, or who ever received ECT. Mdep sample: 5% sample of all Medicare FFS beneficiaries who in the 2-year identification period had major depression, who did receive ECT, had no more than one psychiatric hospitalization, and averaged one medication. management visit every 2–3 months. | Medicare Standard Analytic Files (safs) for 100% of fee-for-service beneficiaries containing all inpatient and outpatient hospital claims. | 690 patients received adjunctive VNS therapy. TRD (4639) and Mdep (7524) patients received other treatment for depression including ECT for the TRD study sample. | VNS therapy involves intermittent stimulation of the left cervical vagus nerve via an implantable pulse generator and lead and an external programming system used for adjusting stimulation settings. | All-cause mortality, suicide attempt or self-inflicted injury, suicide ideation (but not the reduction of those) | Good (4/2/3) | Low |
| Aaronson et al. (2017), USA | To investigate whether adjunctive vagus nerve stimulation (VNS) with treatment as usual in depression has superior long-term outcomes compared with treatment as usual only. | Prospective, open-label, non-randomized, observational registry study | 5 years (01/2006-05/2015) | Patients with a major depressive episode (unipolar or bipolar depression) of at least 2 years’ duration or who had three or more depressive episodes (including the current episode), and who had failed four or more depression treatments (including ECT). In the VNS group 71% were female and the mean age was 48.9 years. In the treatment-as-usual group 70% were female and the mean age was 49.9 years. | Patient registries of 61 sites in the United States specialized in treatment of depression. Postbaseline follow-up visits for all patients were scheduled to occur at 3, 6, 9, 12, 18, 24, 30, 36, 42, 48, 54, and 60 months. | 494 subjects received VNS with treatment-as-usual and 301 subjects received treatment-as-usual only. | No information | All-cause-mortality and suicides | Good (4/2/3) | Moderate |
| **tDCS** | | | | | | | | | |  |
| Chen et al. (2023), China | To directly compare the antidepressant efficiency and safety of longer duration of daily tdcs stimulation as a treatment for MDD. | Randomized, double-blinded, sham-controlled design | 4 weeks | 63 patients with a current acute major depressive episode, ≥17 on the 17-item Hamilton Depression Rating scale (HAMD-17), who where not taking any psychotropic drugs except Z-drugs at least 2 weeks before the first session initiated and during the whole intervention period. The mean age was 28.6 ± 8.8 years, 18 men. | Patients were recruited from the outpatient clinic of Shanghai Mental Health Center. Assessment of depression and anxiety were performed at baseline, at 10 (week 2), and at 12 sessions (week 4) using HAMD-17 score, Concise Health Risk Tracking scale (CHRT) for suicidal ideation and Hamilton Anxiety Scale (HAMA). | 22 patients were allocated to 60-minute sessions, 25 patients to 30 min sessions and 16 patients to sham-stimulation in an initial 2-week (5 sessions per week) randomized controlled trial (RCT) phase. Participants who completed 2 weeks of the RCT phase were eligible to enter a taper phase that consisted of one tDCS session given weekly for two weeks. | Stimulation was performed using a Star-Stim NE multichannel transcranial direct current stimulator. Anode and cathode electrodes were placed over the left DLPFC and right OFC, respectively, with the use of the Omni-Lateral-Electrode system. In a total of 12 sessions that lasted 60 min per session, 2 ma of direct-current stimulation were administered in each session for either 60 min, 30 min, or 1 min (ramp up and down-sham). | The reductive ratio of HAMD-17 score from baseline to week 2 and 4, the change score of Concise Health Risk Tracking scale (CHRT) and Hamilton Anxiety Scale (HAMA) | - | Some concerns |

^a^ The New-castle Ottawa Scale was used for quality assessment of non-randomized studies
^b^ Risk of bias was assessed using Cochrane’s RoB2 tool for randomized trials and ROBINS-I tool for non-randomized studies
ECT: Electroconvulsive therapy, rTMS: Repetitive transcranial magnetic stimulation, VNS: Vagus nerve stimulation, tDCS: Transcranial direct current stimulation
